# Preparation and Evaluation of Atorvastatin-Loaded Nanoemulgel on Wound-Healing Efficacy

**DOI:** 10.3390/pharmaceutics11110609

**Published:** 2019-11-13

**Authors:** Mohamed A. Morsy, Rania G. Abdel-Latif, Anroop B. Nair, Katharigatta N. Venugopala, Amira F. Ahmed, Heba S. Elsewedy, Tamer M. Shehata

**Affiliations:** 1 Department of Pharmaceutical Sciences, College of Clinical Pharmacy, King Faisal University, Al-Ahsa 31982, Saudi Arabia; anair@kfu.edu.sa (A.B.N.); kvenugopala@kfu.edu.sa (K.N.V.); helsewedy@kfu.edu.sa (H.S.E.); tshehata@kfu.edu.sa (T.M.S.); 2 Department of Pharmacology, Faculty of Medicine, Minia University, El-Minia 61511, Egypt; 3 Department of Pharmacology & Toxicology, Faculty of Pharmacy, Minia University, El-Minia 61511, Egypt; dr_raniagalal@yahoo.com; 4 Department of Biotechnology and Food Technology, Durban University of Technology, Durban 4000, South Africa; 5 Department of Histology, Faculty of Medicine, Minia University, El-Minia 61511, Egypt; amirabehery@gmail.com; 6 Department of Pharmaceutics and Industrial Pharmacy, Faculty of Pharmacy, Zagazig University, Zagazig 44519, Egypt

**Keywords:** nanoemulgel, atorvastatin, wound healing

## Abstract

Tissue repair and wound healing are complex processes that involve inflammation, granulation, and remodeling of the tissue. The potential of various statins including atorvastatin (ATR) to improve the wound healing effect was established. The aim of this study was to formulate and evaluate the efficacy of topical application of ATR-based nanoemulgel on wound healing. The prepared formulations (ATR gel, ATR emulgel, and ATR nanoemulgel) were evaluated for their physical appearance, rheological behavior, in vitro drug release and ex vivo drug permeation. The in vivo wound healing effect was evaluated in wound-induced rats. The prepared ATR gel formulations showed good physical properties and were comparable. The release profiles of drugs from gel, emulgel, and nanoemulgel were distinct. Skin permeation potential of ATR was significantly (*p* < 0.05) enhanced when formulated into nanoemulgel. In vivo wound healing studies showed that ATR nanoemulgel exhibited the highest percent wound contraction. Histopathological assessment showed marked improvement in the skin histological architecture after 21 days of ATR nanoemulgel treatment. In conclusion, the data demonstrated here signify the prospective of ATR nanoemulgel as an innovative therapeutic approach in wound healing.

## 1. Introduction

Wound healing is a dynamic and complex biological process which requires orchestration of different cellular processes to help damaged skin restores its normal function and structure [1]. The healing process of the open wounds includes interdependent and overlapping stages of hemostasis, inflammation, proliferation, revascularization, and remodeling [2]. These phases must occur in an integrated sequence and in optimal intensity to properly allow wound healing [3]. Therefore, developing new treatment modalities for wound healing is highly required; especially as current medical therapies for wound care are limited.

Statins are anti-hyperlipidemic agents that are widely used in patients to prevent cardiovascular events [4]. Recently, statins have shown efficacy in the treatment of a wide variety of dermatologic disorders, such as urticaria [5], psoriasis [6], and acne [7]. Because of their diverse pleiotropic effects, statins have been also suggested to be useful in wound healing as they can modulate cellular processes including inflammation, apoptosis, and proliferation [8]. Besides, they are reported to decrease oxidative stress and improve endothelial as well as microvascular functions which may have a role in accelerating and enhancing healing processes [9,10]. Indeed, various studies have evaluated the healing efficacy of different statins, including simvastatin, atorvastatin (ATR), and pravastatin, on various skin wounds [8,11,12]. The most consistent results in wound healing came with ATR—a second-generation synthetic statin—which was shown to have specific properties such as long half-life, active metabolites, lipophilicity, and high protein binding [13,14].

Due to the multiple side effects of oral statins, including myopathy and liver toxicity [15], topical drug delivery system is suggested to be a reasonable alternative to oral statin in wound healing, as it provides better drug delivery with prolonged action and less side effects [16]. Transdermal drug delivery systems include transdermal patches, gels, and emulgels [17,18,19]. Recently, nanoemulgel—a novel approach for topical delivery of hydrophobic drugs—has been reported to have several favorable properties including improved physical stability, non-toxicity, and a non-irritant nature [10,20]. Apart from these properties, it exhibits a dual release control system—hydrogel and nanoemulsion—and has nano-sized particles which allow rapid permeation and delivery of active pharmaceutical ingredients [10,21]. The previously mentioned characteristics of the nanoemulgel provide improved drug efficacy compared to other traditional formulations in treatment of various skin diseases as well as bacterial and fungal infections [21,22,23]. The present study was conducted to develop different topical formulations of ATR using gel, emulgel, and nanoemulgel systems. Additionally, the study aimed to evaluate the wound healing efficacy of ATR nanoemulgel compared to other gel formulations on excision wound-induced rats.

## 2. Materials and Methods

### 2.1. Materials

Calcium salt of ATR and sodium salt of carboxymethyl cellulose (CMC) were gift samples from Saudi Pharmaceutical Industries & Medical Appliances Corporation ((SPIMACO), Riyadh, Saudi Arabia). Tween 80 and propylene glycol were purchased from Sigma-Aldrich Corp. (St. Louis, MO, USA). All other chemicals and solvents were of high analytical grade and were obtained from El-Nasr Pharmaceutical Chemicals Co. (Qaliubiya, Egypt).

### 2.2. Preparation of Topical ATR Formulations

#### 2.2.1. Preparation of Gel

Aqueous polymeric gel base was prepared by adding Na-CMC (1% *w/w*) in water (up to 50 mL) and stirring continuously on a magnetic stirrer (Jeio Tech TM-14SB, Medline Scientific, Oxfordshire, UK) until the gel was formed. Then required amount of ATR (2.5% *w/w*) was added slowly to the polymeric gel under constant stirring to get a homogenous dispersion.

#### 2.2.2. Preparation of Emulgel

Primary emulsion was prepared by adding the required amount of the drug in 12.5 mL of the liquid paraffin-containing Tween 80 (1 mL; emulsifier) and propylene glycol (1 mL; coemulsifier) and vortexed (2000 rpm for 5 min). Water (20 mL) was slowly added to the previous mixture, while vortexing for 10 min. The polymeric gel base was prepared in the remaining water. The drug-loaded emulsion was slowly added to the polymeric gel base and then mixed with a mixer (Heidolph RZR 1, Heidolph Instruments, Schwabach, Germany) for 5 min until a homogenous emulgel was obtained [24].

#### 2.2.3. Preparation of Nanoemulgel

Primary emulsion containing ATR was formulated as in the emulgel preparation, followed by homogenization (T 25 digital Ultra-Turrax, IKA, Staufen, Germany) for 5 min at 10,000 rpm and then sonication (XL-2000, Qsonica, Newtown, CT, USA) for 10 min to produce nanoemulsion. The polymeric gel base was prepared in the remaining water. The drug-loaded nanoemulsion was slowly added to the polymeric gel base and then mixed with a mixer for 10 min until a homogenous nanoemulgel was obtained. Compositions of various gel formulations are summarized in Table 1.

### 2.3. Physical Examination

The prepared gels were inspected visually for their color, appearance, and homogeneity. The pH of the gel formulations was tested at room temperature (MW802, Milwaukee Instruments, Szeged, Hungary).

#### 2.3.1. Spreadability Test

Spreadability of the gel formulations determines their bioavailability efficiency as it shows the extent of area to which the formulation could readily spread on application to skin or the affected part. The spreadability value of different gel formulations was determined by measuring the spreading diameter of 1 g of gel between two horizontal glass plates (25 cm × 25 cm) under certain load after 1 min [25].

#### 2.3.2. Rheological Studies

The viscosity of the gel formulations was tested at room temperature during handling and storage. Viscosity of the different gels was determined using DV-II+PRO viscometer (Brookfield, Middleboro, MA, USA). The dynamic viscosity measurements were made using spindle R5 rotated at 0.5 rpm. Samples of the gels were allowed to settle over 30 min at the assay temperature (25 ± 1 °C) before the measurements were taken.

#### 2.3.3. Particle Characterization

Ten microliters of different gel preparations were diluted with 3 mL of distilled water and the particle size and polydispersity index were measured by Zetasizer Nano (Malvern Panalytical, Worcestershire, UK). The morphology of the nanoemulgel particles was investigated with a scanning electron microscope (JEOL JSM-5510LV, Tokyo, Japan). The nanoemulgel was diluted with water (1:10). One drop of the diluted sample was mounted on a stub covered with double adhesive tape and coated with gold after drying for visualization.

### 2.4. In Vitro Drug Release Study

In vitro release of ATR from different preparations was determined by a simple dialysis method using a dissolution-dialysis apparatus, the cell of which was developed in our laboratory. The dissolution cell consisted of a hollow glass cylinder (length of 15 cm and internal diameter of 2.9 cm). The backing membrane reservoirs of gels (donor) were attached to the glass tubes at the end of the internal diameter of 2.9 cm and covered with semipermeable membranes with the aid of rubber bands. The tubes were attached to the dissolution apparatus. Weighed quantity of gels (250 mg) equivalent to 6.25 mg of ATR was taken in the tube and then the tubes were allowed to stir at 50 rpm in 300 mL phosphate buffer (pH 7.4) maintained at 37 ± 0.5 °C. This volume provided complete sink conditions for the drug. About 2 mL samples were withdrawn at specified time intervals (0.5, 1, 2, 3, 4, 5, and 6 h) and replaced with equal volumes of fresh buffer solution to keep the volume constant during the experiment. Samples were analyzed spectrophotometrically (Jenway 6305 spectrophotometer, Jenway, Staffordshire, UK) at 241 nm [24]. The drug release profile was used to determine the correlation coefficient (r^2^) and release kinetics of gel formulations using various mathematical models [26]:

a. Zero order model *Q = Q_0_ + kt*;
b. First order model *Q = Q_0_ × e^kt^*;
c. Higuchi model *Q = k × t*^0.5^;
d. Korsmeyer-Peppas model *Q = k × t^n^*;

where *Q* represents quantity of drug released in time *t*, *Q_0_* represents value of *Q* at zero time, *k* represents the rate constant and *n* represents the diffusional exponent. The model which showed the highest correlation coefficient (r^2^) value for the drug release data was considered as the best fit.

### 2.5. Preliminary Stability Testing

The prepared formulations were kept in tightly closed plastic jars and stored at a relative humidity of 60% and temperature of 4 °C over a period of 6 months. The physical characteristics as well as in vitro release data of ATR from the stored formulations were evaluated.

### 2.6. Ex Vivo Evaluation of ATR Release (Permeation Study)

#### 2.6.1. Preparation of Rat Skin

Rat skin was used in ex vivo permeation studies due to various advantages like its availability, ease of handling, small size, low cost, and structural similarity to the human skin [27]. Though more permeable than human skin, the influence of physicochemical properties on skin transport can be effectively determined using the rat skin [28].

Wistar rats (250–300 g) were obtained from the animal breeding center, College of Science, King Faisal University. Hair was removed from the abdominal skin using an electric clipper. After sacrifice, rats were fixed and a full-thickness of rat skin was excised from the abdominal region. The adipose tissue was removed and the excised skin was hydrated using phosphate buffer (pH 7.4; containing 0.02% sodium azide as a preservative) at 4 °C overnight [24].

#### 2.6.2. Permeation of ATR from Different Formulations

The ex vivo permeation of ATR from different preparations was determined using previously described diffusion cells [29]. The skin membranes were mounted in the diffusion cell, where the stratum corneum side faced the donor (drug-loaded system) and the dermal side faced the receptor compartment which contained 100 mL phosphate buffer (pH 7.4) and 0.02% sodium azide at 37 ± 0.5 °C. Then, 0.2 g of each tested formulations were placed separately into membrane holders and fixed to the glass tubes; skin membranes were used to cover the preparations with the stratum corneum side face. The tubes were attached to the dissolution apparatus with Parafilm (Bemis, Oshkosh, WI, USA) to avoid water evaporation, then they were allowed to stir at 100 rpm. At 0.5, 1, 2, 3, 4, 5, and 6 h after starting the experiment, 1 mL aliquots were sampled from the receptor compartment with the fresh buffer replacement. Samples were analyzed spectrophotometrically (Jenway 6305 spectrophotometer, Jenway, Staffordshire, UK) at 241 nm. Samples collected from permeation of drug-free systems were used as a blank [30]. Ex vivo permeation parameters including steady state transdermal flux (SSTF), lag time, and enhancement ratio (ER) for percutaneous absorption of ATR across rat skin were estimated for different formulations.

SSTF = amount of permeated drug/(area × time);
ER = SSTF from test/SSTF from control.

### 2.7. In Vivo Evaluation of Wound Healing Efficiency

#### 2.7.1. Experimental Design

The study was conducted on adult male Wistar rats of 200–250 g. Animals were housed under a specific pathogen-free condition with a 12-h day/night cycle and 25 ± 1 °C temperature. Besides, they were fed standard pellet chow and permitted free access to tap water. The ex vivo and in vivo experiments were approved (273/9/2019; dated August 9, 2019) by the Research Ethics Committee, Faculty of Medicine, Minia University, Egypt and were performed in accordance with the animal research ethical standards of the Research Ethics Committee, King Faisal University and in compliance with the guidelines of the National Committee of Bioethics (NCBE), KACST, Saudi Arabia. 

The rats were anesthetized using an intraperitoneal injection of ketamine hydrochloride (50 mg/kg) and xylazine (10 mg/kg). After anesthesia, the dorsal region was shaved and then sterilized using an alcohol swab. Using an autoclave-sterilized razor-sharp metal punch, a full-thickness wound of 1-cm diameter was performed in the back of each animal. The location of the wound prevented the rats from accessing the wounds and reopening them. Animals then were randomly divided into three groups (*n* = 6) and were individually caged. Group 1 received ATR gel, group 2 received ATR emulgel, and group 3 received ATR nanoemulgel. All gel preparations were applied topically on the wound area on a daily basis from the day of creation of the excision wound for the entire experiment time. Wounds were left air-exposed and wound healing ratio was evaluated on days 0, 7, 14, and 21 of the experiment.

#### 2.7.2. Quantification of Wound Area

On days 0, 7, 14, and 21 of the experiment, lesions were photographed with a digital camera. Lesion area at the pre-identified times was measured digitally using the software ImageJ, version 1.45 (freeware; rsbweb.nih.gov/ij; Bethesda, MD, USA). The lesion areas obtained from the pictures were used to calculate percentage wound area.

### 2.8. Skin Irritation Studies

Adult male Wistar rats weighing 200–250 g were used for skin irritation testing. The animals were maintained under standard conditions, fed standard pellet chow, and permitted free access to tap water. Each tested formulation was applied on the hair-free skin of rats by uniform spreading over an area of 4 cm^2^ on their backs. The skin surface was observed for any sensitivity reaction such as erythema (redness) or edema after 24, 48, and 72 h of the formulation application. The observed sensitivity reaction was scored either 0, 1, 2, or 3 for no reaction, slight erythema, moderate and patchy erythema, and severe erythema with or without edema, respectively [31].

### 2.9. Histologic Evaluations

On days 0 and 21 after lesion induction, animals were anesthetized and then fixed to the operating table. The wound and its surrounding healthy skin including fascia muscles was excised. The samples were fixed with 10% neutral buffered formalin then embedded in paraffin, sliced into 4-µm-thick sections using a microtome, and stained with hematoxylin-eosin (H&E). Using light microscopy, the specimens were assessed by an independent investigator for the severity of histopathologic changes and healing status. Wound healing was assessed according to Hazrati scoring of four histological parameters including re-epithelialization, granulation tissue, inflammatory cells, and angiogenesis. The total healing score in each case was calculated by adding the scores of individual criteria, with lower scores indicating poorer wound healing. Healing status was graded as good (16–20), fair (11–15), and poor (5–10) [32].

### 2.10. Statistical Analysis

Results were expressed as mean ± SD. Statistically significant differences between groups were detected through one-way ANOVA followed by Tukey’s post-analysis test for group comparisons. Statistical analysis was performed using GraphPad Prism software version 5.00 (San Diego, CA, USA). The level of *p* < 0.05 was considered statistically significant for all tests.

## 3. Results

### 3.1. Physical Examination

**Figure 1:**
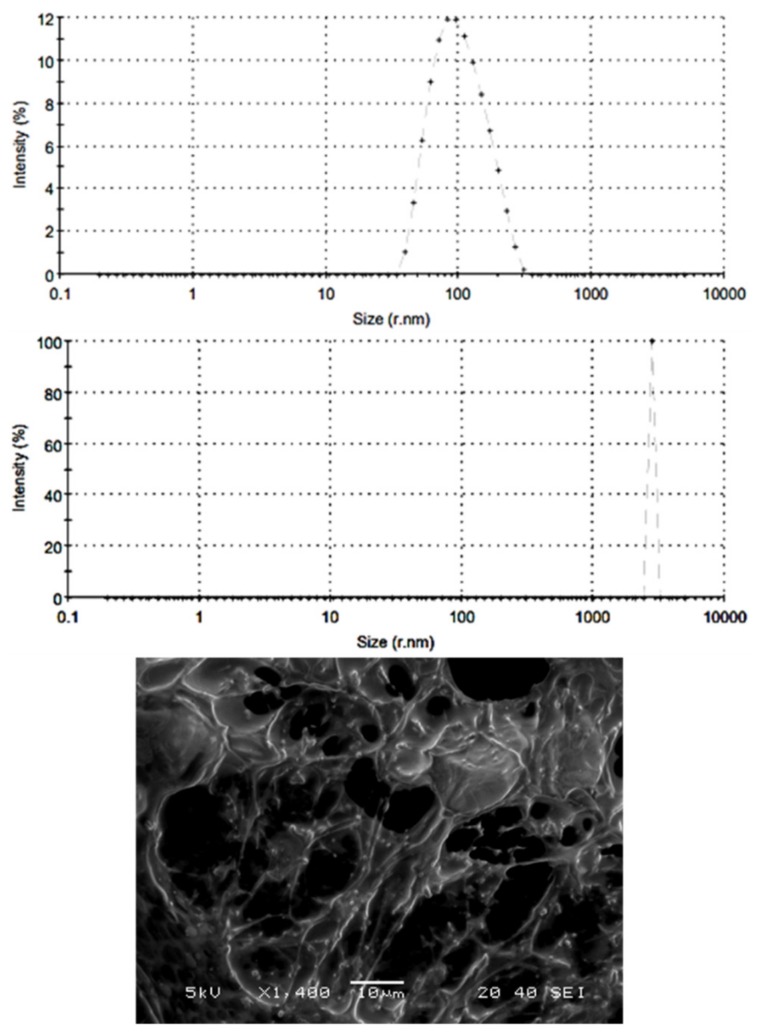
Size distribution and surface morphology. Upper panel: atorvastatin nanoemulgel. Middle panel: atorvastatin emulgel. Lower panel: scanning electron microscopic image of nanoemulgel.

Table 2 displayed the physical properties of different prepared formulations. The pH values (7.6–7.8) of gel formulations were comparable and were not likely to cause any irritations to the skin after application. All gel formulations showed good homogeneity. The physical appearance of the tested formulations was white opaque. Data showed spreadability values of 66 ± 0.88, 54 ± 0.95, and 51 ± 0.66 mm for fresh ATR gel, emulgel, and nanoemulgel formulations, respectively. The results showed that nanoemulgel formulation of ATR was more viscous (85,900 ± 2050 cp) (*p* < 0.05) as compared with the gel formulation (58,500 ± 450 cp). Furthermore, the viscosity of nanoemulgel was significantly lower than emulgel (97,250 ± 1150 cp). The particle size determination showed a good distribution with polydispersity index value less than 0.3 for both ATR emulgel and nanoemulgel preparations with spherical particle sizes of 3082 nm and 148 nm, respectively (Figure 1).

### 3.2. In Vitro Release of the Drug

**Figure 2:**
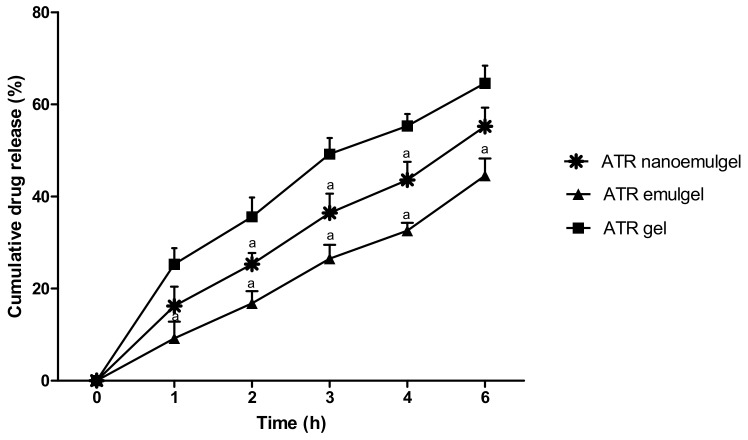
Release of atorvastatin (ATR) from different gel formulations across semipermeable membrane into phosphate buffer (pH 7.4) at 37 °C. Values are expressed as mean ± standard deviation (SD) and were analyzed by one-way ANOVA followed by Tukey’s multiple comparisons test. ^a^ *p* < 0.05 compared to ATR gel.

It was observed that the percentage of the drug released from CMC gel was higher when compared to emulgel and nanoemulgel formulations (Figure 2). About 65% of ATR was released from CMC gel across the semipermeable cellulose membranes after 6 h, while only 55% and 44% of the drug was released at the same time from CMC nanoemulgel and emulgel, respectively. Consequently, rank order of the various gel formulations based on their maximum drug release is ATR gel > ATR nanoemulgel > ATR emulgel. It is evident from Table 3 that the release kinetics of ATR from all gel formulations fitted into the Higuchi diffusion model. Therefore, the predominant mechanism for ATR release from these gels is diffusion.

### 3.3. Stability Study of ATR-Loaded Formulations

Under each storage condition of 60% relative humidity and 4 °C temperature for a period of 6 months, ATR release and the physical characteristics of all investigated formulations were evaluated. The results showed that there was no evident change in the color, appearance, spreading, or viscosity after subjecting the tested formulations to stability studies (Table 2). In addition, no significant change was observed in drug release for the stored formulations compared to their comparable fresh preparations (data are not shown).

### 3.4. Ex Vivo Study (Permeation Study)

**Figure 3:**
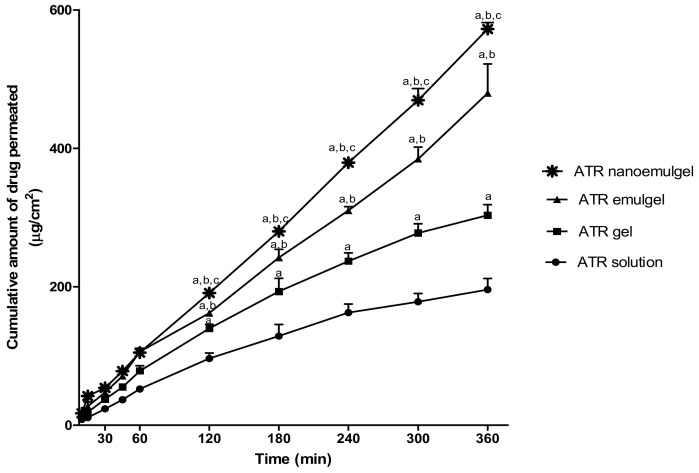
Permeation profiles of atorvastatin (ATR) through the excised rat skin from different gel formulations compared to solution (control). Values are expressed as mean ± standard deviation (SD) and were analyzed by one-way ANOVA followed by Tukey’s multiple comparisons test. ^a,b,c^ *p* < 0.05 compared to ATR solution, ATR gel, and ATR emulgel, respectively.

Figure 3 shows the order of ATR permeation through the excised rat skin from gel, emulgel, and nanoemulgel ATR formulations compared to ATR solution that was used as a control formulation. Tested formulation showed a rank order of ATR permeation as the following: ATR nanoemulgel > ATR emulgel > ATR gel > ATR solution. It is also evident from Figure 3 that the cumulative amount of ATR permeated (µg/cm^2^) was significantly higher (*p* < 0.05) in ATR nanoemulgel after 2 h, as compared to ATR emulgel, gel, and solution. Data of the present study revealed that gel formulation of ATR could significantly enhance its permeability by 1.55 fold compared to ATR solution. Additionally, the SSTF of ATR from ATR gel was significantly higher than SSTF value of ATR solution (51.89 ± 1.71 vs. 34.39 ± 1.18 µg/cm^2^·h, respectively) (Table 4). CMC emulgel formulation could significantly (*p* < 0.05) enhance skin permeation parameters of ATR compared to CMC gel formulation. Results showed that SSTF value of ATR emulgel was 76.11 ± 3.47 µg/cm^2^·h with 2.45-fold enhancement ratio and short lag time of 6.94 ± 1.77 min. Moreover, ATR nanoemulgel showed the highest enhancement ratio of drug permeability (2.92 fold) with the highest SSTF value of 95.39 ± 1.55 µg/cm^2^·h, compared to ATR solution. On the other hand, ATR nanoemulgel exhibited the shortest lag time (3.31 ± 1.33 min) compared to all formulations under investigation.

### 3.5. In Vivo Wound Healing Efficiency

**Figure 4:**
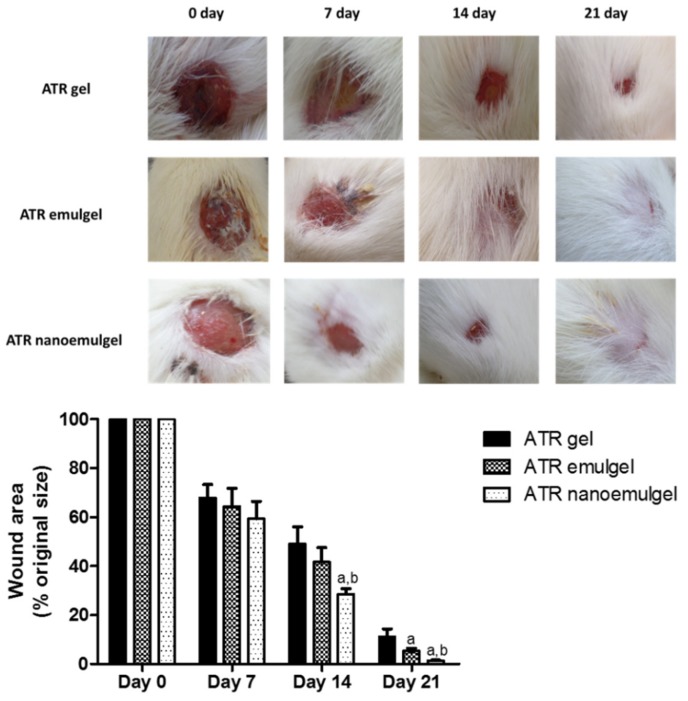
Upper panel: Macroscopic wound closure in rats that received topical atorvastatin (ATR) gel, ATR emulgel, and ATR nanoemulgel. Representative pictures showing the time course of wound healing in rats on days 0, 7, 14, and 21. Lower panel: Graphical presentation of changes in wound area in rats received topical ATR gel, ATR emulgel, and ATR nanoemulgel. Changes in wound area are expressed as a percentage of the initial wound area. Values represent mean ± standard deviation (SD) of six animals per group and were analyzed by one-way ANOVA followed by Tukey’s multiple comparisons test. ^a,b^ *p* < 0.05 compared to ATR gel and ATR emulgel, respectively, at the same day.

Macroscopic evaluation was performed to monitor wound healing through photographs. Figure 4 (upper panel) shows representative pictures of the regression of the wound area throughout the experiment. The wound area, calculated as a percentage of the initial wound area, was determined for all animal groups (Figure 4; lower panel). On days 14 and 21 of the experiment, it was observed that rats that received ATR nanoemulgel showed a significant reduction in wound area compared to other groups that received either ATR gel or emulgel.

### 3.6. Skin Irritation Testing

All tested formulations showed a sensitivity reaction of score 0 upon their application on the hair-free skin of rats’ backs. The applied area was observed for 3 days and no erythema, edema, or irritation were recorded during the whole period of study.

### 3.7. Histopathological Assessment

**Figure 5:**
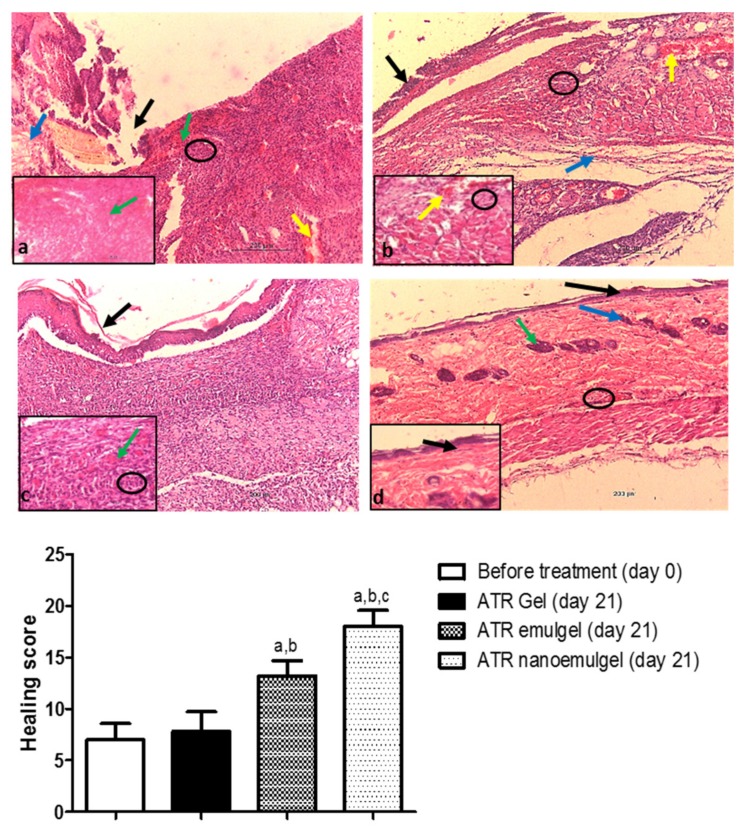
Upper panel: Photomicrographs of the rat skin tissue. (**a**) Wound area before treatment at day 0. Absence of epithelization in the epidermal layer (black arrow). Dermal layer shows loss of normal arrangement of collagen fibers (blue arrow). Severe congestion (green arrows), hemorrhage (yellow arrow), and inflammatory cell infiltrations (black circle) are observed. (**b**) Atorvastatin (ATR) gel-treated group at day 21. Partial epithelialization (black arrow) is exhibited. Dermal layer has granulation tissue (blue arrow) with vascular congestion and hemorrhage (yellow arrows) and mild inflammatory cell infiltrations (black circles). (**c**) ATR emulgel-treated group at day 21 has complete epithelization with formation of thin keratin layer (black arrow). The underlying dermal layer shows vascular granulation tissues in addition to fibrous connective tissues (green arrow) and inflammatory cell infiltrations (black circle). (**d**) ATR nanoemulgel-treated group at day 21. Complete epithelization of the epidermis (black arrows) with presence of skin hair follicles (blue arrow) and sebaceous glands (green arrow) are exhibited. There are few inflammatory cell infiltrations (black circle). (H&E ×200 µm and inset ×50 µm). Lower panel: A semi-quantitative analysis of healing status in rats at day 0 before the treatment and after 21 days of receiving topical ATR gel, ATR emulgel, and ATR nanoemulgel. Healing status is assessed according to healing score. Values are expressed as mean ± as mean ± standard deviation (SD) of five readings and were analyzed by one-way ANOVA followed by Tukey’s multiple comparisons test. ^a,b,c^ *p* < 0.05 compared to non-treated, ATR gel-treated, and ATR emulgel-treated groups, respectively.

Before treatment with ATR gel, the wound area showed severe congestion, hemorrhage, and inflammatory cells’ infiltration, with absence of epithelium and normal collagen disposition (Figure 5a, upper panel). After 21 days of ATR gel treatment, a smaller number of inflammatory cells was observed with partial epithelialization (Figure 5b, upper panel). The rat group that received ATR gel showed poor healing with a healing score of 7.8 ± 1.9 (Figure 5, lower panel). After 21 days of ATR emulgel treatment, the wound area exhibited complete epithelization with the formation of a thin keratin layer. Granulation and fibrous connective tissues were also observed in underlying dermal layer (Figure 5c, upper panel). Healing status after ATR emulgel was fair and showed a healing score of 13.2 ± 1.4 (Figure 5, lower panel). Figure 5d (upper panel) shows a marked improvement in the skin histological architecture with good healing status after 21 days of ATR nanoemulgel treatment. No indication for the presence of wound in this area is due to epithelialization of the epidermis of skin. The wound area showed a high healing score of 18 ± 1.5 (Figure 5 lower panel). The epidermis was normal and the dermis contained normal skin appendages.

## 4. Discussion

For wound healing purposes, several studies have shown the effectiveness of ATR in restoring endothelial function and accelerating tissue repair of a skin lesion in both animal and human studies [8,33]. However, the low solubility and bioavailability of ATR [34,35] may limit its maximal benefits in wound healing. In this regard, this study was designed to evaluate the effectiveness of alternative topical preparations of ATR in wound healing using nanoemulgel in comparison to other conventional ATR preparations of gel and emulgel.

Owing to their high aqueous component, gels as a dosage form generally allow a greater dissolution of the drug and permits easy migration of it through a vehicle [36]. Indeed, the results of the present study showed that ATR gel exhibited the highest release of the drug during the in vitro release study. On the contrary, ATR emulgel and nanoemulgel showed significantly (*p* < 0.05) low release of the drug during the same study. This result can be explained by the higher viscosity of emulgel and nanoemulgel as compared to ATR gel formulation. The higher viscosity of the vehicle was associated with increased resistance to the drug molecules’ mobility and diffusion [37]. Additionally, the low release rate of ATR from the emulgel and nanoemulgel could be attributed to the low water content of the solvent mixtures and incorporation of liquid paraffin oil [38]. On the other hand, both ATR emulgel and nanoemulgel showed high permeation compared to ATR gel in ex vivo permeation study. The enhanced permeation effect of ATR from emulgel and nanoemulgel could be attributed to their surfactant contents that act as permeability enhancers in addition to the colloidal properties.

The higher viscosity of ATR emulgel and nanoemulgel can also markedly affect the spreadability value of the formulation. The study of Majithiya et al. showed that the greater the viscosity is, the lesser the spreadability and greater the retention of emulgel on the skin are [39]. This explanation was supported by our results that showed lower spreadability values of ATR emulgel and nanoemulgel compared to that of ATR gel.

Emulgel has merits of both gel and emulsion; it behaves as a reservoir of drugs as emulsion droplets allow encapsulation of hydrophobic drugs and hasten its skin permeation [40]. Nanoemulgel is prepared by developing nanoemulsion with droplet size range <200 nm to be incorporated into a hydrogel matrix [41,42]. In the present study, ATR is formulated as oil-in-water (o/w) nanoemulsion in a gel phase with average particle size of 148 nm.

The current data showed that both ATR emulgel and nanoemulgel exhibited greater permeation through the rat skin compared to ATR gel. This could be attributed to the nonionic surfactant (Tween 80) interaction with rat skin lipids, which could increase the fluidity of skin membranes resulting in an enhancing drug diffusion rate across skin layers [43]. However, compared to ATR emulgel, results showed that ATR nanoemulgel had better permeation. One possible reason that could have an effect on the enhanced permeation of ATR nanoemulgel is the nano droplets of nanoemulsion, which gave the highest surface area for ATR permeation and for releasing a high drug concentration on the affected area [44].

Additionally, the reduced lag time to drug permeation reported in ATR nanoemulgel may be attributed to the mucoadhesive property of hydrogel. The mucoadhesive properties of hydrogel have a pivotal role in increasing the contact period of the drug over the skin [45]; better contact with the skin allows the effect to appear sooner, resulting in short lag times.

On contrary to the results of the ex vivo permeation study, the rank order of maximum drug release in the in vitro study was ATR gel > ATR nanoemulgel > ATR emulgel. The discrepancy in the rank order of drug release in ex vivo and in vitro studies may be attributed to the different characteristics of cellophane membrane and animal skin [46].

Stability studies of the investigated ATR formulations concluded that the drug does not undergo degradation in storage. Additionally, no adverse effects were observed in animal skin treated with ATR gel, emulgel, or nanoemulgel. These results indicated that the investigated ATR gel, emulgel, and nanoemulgel have good formulation and skin tolerability, which are advantageous in increasing patient acceptability to the treatment. Consequently, the investigated formulations are eligible for further in vivo investigations.

Besides their anti-inflammatory effect, statins can modulate cellular processes, such as apoptosis, proliferation, and migration, which makes them promising therapy for wound healing [12,16]. Indeed, several studies have tested the effectiveness of statins in the treatment of skin diseases and wounds using ATR of different formulations and different routes of administration including oral gavage, intraperitoneal injection, and topical application [8,38,47]. However, the current study is the first study to explore the effectiveness of ATR in wound healing using nanoemulgel as a novel technique for topical drug delivery, compared to other conventional topical preparations, namely gel and emulgel. Data of the present in vivo study illustrated that the ATR nanoemulgel-treated group exhibited a significant decrease in wound area during the experiment period compared to both ATR gel- and ATR emulgel-treated groups. This greater wound contraction may be ascribed to the dual effect of the drug and nanoemulgel system which allows better delivery of hydrophobic drugs [48]. Due to its good adhesion properties, studies showed that the nanoemulgel system exhibits a larger concentration gradient across the skin, which leads to better skin penetration and consequently good healing properties [45,49]. Further, ATR nanoemulgel-treated rats showed marked improvement in pathological changes of the wound tissue as compared to other treatment groups. Results from H&E stained skin sections of the ATR nanoemulgel-treated group revealed complete epithelial regeneration and a well-structured layer that covered the entire area of the wound. By the end of the experiment, it was noticed that treatment with ATR nanoemulgel provided almost complete wound closure and that the wound area was completely covered with hair.

The major concern in the treatment of chronic wounds is the lack of effective therapeutic options for clinical use. The gel-based formulations including emulgel may also face issues such as short residence time in the application site. However, the nanoemulgel formulation with greater retention in the affected area can release the drug steadily and could subsequently help in bathing the skin surface for an extended period, which in turn can accelerate wound healing.

## 5. Conclusions

Taking all of the above into account, it can be concluded that ATR nanoemulgel showed the highest wound healing power with complete wound closure and epithelization within 21 days. Therefore, the current study may provide an innovative approach for efficient wound healing using ATR nanoemulgel topical application. Further experimental and clinical studies are needed to confirm the results of the present study.

## Figures and Tables

**Table 1:** Compositions of gel, emulgel, and nanoemulgel of 2.5% (*w/w*) atorvastatin.

Formulation	Atorvastatin (% *w/w*)	Carboxymethyl Cellulose (% *w/w*)	Liquid Paraffin (mL)	Tween 80 (mL)	Propylene Glycol (mL)	Water Up To (mL)
Gel	2.5	1	-	-	-	50
Emulgel	2.5	1	12.5	1	1	50
Nanoemulgel	2.5	1	12.5	1	1	50

**Table 2:** Physical examinations of the fresh formulations and following 6 months storage at a relative humidity of 60% and temperature of 4 °C.

Properties		ATR Gel	ATR Emulgel	ATR Nanoemulgel
**Color**	Fresh	White, Opaque	White, Opaque	White, Opaque
	Stored	White, Opaque	White, Opaque	White, Opaque
**Homogeneity**	Fresh	Homogenous	Homogenous	Homogenous
	Stored	Homogenous	Homogenous	Homogenous
**Spreadability (mm)**	Fresh	66 ± 0.88	54 ± 0.95 ^a^	51 ± 0.66 ^a^
	Stored	61 ± 0.60	52 ± 0.55 ^a^	49 ± 0.25 ^a^
**Viscosity** **(cp)**	Fresh	58,500 ± 450	97,250 ± 1150 ^a^	85,900 ± 2050 ^a,b^
	Stored	61,350 ± 850	101,550 ± 2250 ^a^	97,500 ± 3150 ^a,b^

Values are expressed as mean ± standard deviation (SD) and were analyzed by one-way ANOVA followed by Tukey’s multiple comparisons test. ^a,b^ *p* < 0.05 compared to ATR gel and ATR emulgel, respectively. ATR: atorvastatin.

**Table 3:** Release kinetics of different atorvastatin (ATR) formulations.

Mathematical Models	ATR Gel	ATR Emulgel	ATR Nanoemulgel
Zero order	0.943	0.989	0.979
First order	0.879	0.901	0.906
Higuchi	0.983	0.994	0.995
Korsmeyer-Peppas	0.975	0.988	0.989

**Table 4:** Ex vivo skin permeation parameters of different atorvastatin (ATR) formulations.

Time	ATR Sol	ATR Gel	ATR Emulgel	ATR Nanoemulgel
**SSTF (µg/cm^2^·h)**	34.39 ± 1.18	51.89 ± 1.71 ^a^	76.11 ± 3.47 ^a,b^	95.39 ± 1.55 ^a,b,c^
**Lag time (min)**	24.59 ± 5.48	21.08 ± 4.53	6.94 ± 1.77 ^a,b^	3.31 ± 1.33 ^a,b^
**ER (folds)**	1.00	1.55 ^a^	2.45 ^a,b^	2.92 ^a,b,c^

Values are expressed as mean ± standard deviation (SD) and were analyzed by one-way ANOVA followed by Tukey’s multiple comparisons test. ^a,b,c^ *p* < 0.05 compared to ATR sol, ATR gel, and ATR emulgel, respectively. Sol, solution; SSTF, steady state transdermal flux; ER, enhancement ratio.

## References

[1] Eming S.A., Martin P., Tomic-Canic M. (2014). Wound repair and regeneration: Mechanisms, signaling, and translation. Sci. Transl. Med..

[2] Landén N.X., Li D., Ståhle M. (2016). Transition from inflammation to proliferation: A critical step during wound healing. Cell. Mol. Life Sci..

[3] Reinke J., Sorg H. (2012). Wound repair and regeneration. Eur. Surg. Res..

[4] Mooradian A.D. (2019). Evidence-Based Cardiovascular Risk Management in Diabetes. Am. J. Cardiovasc. Drug.

[5] El-Korashi L., Soliman M., Attwa E., Mohamed N. (2018). Role of Atorvastatin in Treatment of Chronic Spontaneous Urticaria Patients: A Controlled Clinical Trial. Egypt. J. Immunol..

[6] Ramessur R., Gill D. (2017). The effect of statins on severity of psoriasis: A systematic review. Indian J. Dermatol. Venereol. Leprol..

[7] Ahmadvand A., Yazdanfar A., Yasrebifar F., Mohammadi Y., Mahjub R., Mehrpooya M. (2018). Evaluating the effects of oral and topical simvastatin in the treatment of acne vulgaris: A double-blind, randomized, placebo-controlled clinical trial. Curr. Clin. Pharmacol..

[8] Suzuki-Banhesse V.F., Azevedo F.F., Araujo E.P., do Amaral M.E., Caricilli A.M., Saad M.J., Lima M.H. (2015). Effect of atorvastatin on wound healing in rats. Biol. Res. Nurs..

[9] Zhou Q., Liao J.K. (2010). Pleiotropic effects of statins. Circ. J..

[10] Choudhury H., Gorain B., Pandey M., Chatterjee L.A., Sengupta P., Das A., Molugulu N., Kesharwani P. (2017). Recent update on nanoemulgel as topical drug delivery system. J. Pharm. Sci..

[11] Asai J., Takenaka H., Hirakawa S., Sakabe J.-i., Hagura A., Kishimoto S., Maruyama K., Kajiya K., Kinoshita S., Tokura Y. (2012). Topical simvastatin accelerates wound healing in diabetes by enhancing angiogenesis and lymphangiogenesis. Am. J. Pathol..

[12] Bracht L., Caparroz-Assef S.M., Magon T.F.d.S., Ritter A.M.V., Cuman R.K.N., Bersani-Amado C.A. (2011). Topical anti-inflammatory effect of hypocholesterolaemic drugs. J. Pharm. Pharmacol..

[13] Neuvonen P.J., Niemi M., Backman J.T. (2006). Drug interactions with lipid-lowering drugs: Mechanisms and clinical relevance. Clin. Pharmacol. Ther..

[14] Lennernäs H. (2003). Clinical pharmacokinetics of atorvastatin. Clin. Pharmacokinet..

[15] Bellosta S., Corsini A. (2018). Statin drug interactions and related adverse reactions: An update. Expert Opin. Drug Saf..

[16] Farsaei S., Khalili H., Farboud E.S. (2012). Potential role of statins on wound healing: Review of the literature. Int. Wound J..

[17] Kumar L., Verma R. (2010). In vitro evaluation of topical gel prepared using natural polymer. Int. J. Drug Deliv..

[18] Malvey S., Rao J.V., Arumugam K.M. (2019). Transdermal drug delivery system: A mini review. Pharma Innov..

[19] Gul R., Ahmed N., Ullah N., Khan M.I., Elaissari A. (2018). Biodegradable ingredient-based emulgel loaded with ketoprofen nanoparticles. AAPS Pharm. Sci. Tech..

[20] Jaiswal M., Dudhe R., Sharma P. (2015). Nanoemulsion: An advanced mode of drug delivery system. 3 Biotech..

[21] Mahtab A., Anwar M., Mallick N., Naz Z., Jain G.K., Ahmad F.J. (2016). Transungual delivery of ketoconazole nanoemulgel for the effective management of onychomycosis. AAPS Pharm. Sci. Tech..

[22] Gupta A., Bonde S.R., Gaikwad S., Ingle A., Gade A.K., Rai M. (2013). Lawsonia inermis-mediated synthesis of silver nanoparticles: Activity against human pathogenic fungi and bacteria with special reference to formulation of an antimicrobial nanogel. IET Nanobiotechnol..

[23] Alam M.S., Ali M.S., Alam N., Siddiqui M.R., Shamim M., Safhi M. (2013). In vivo study of clobetasol propionate loaded nanoemulsion for topical application in psoriasis and atopic dermatitis. Drug Invent. Today.

[24] Ibrahim M., Shehata T. (2012). The enhancement of transdermal permeability of water soluble drug by niosome-emulgel combination. J. Drug Deliv. Sci. Technol..

[25] Dantas M.G., Reis S.A., Damasceno C.M., Rolim L.A., Rolim-Neto P.J., Carvalho F.O., Quintans-Junior L.J., Almeida J.R. (2016). Development and evaluation of stability of a gel formulation containing the monoterpene borneol. Sci. World J..

[26] Shah J., Nair A.B., Jacob S., Patel R.K., Shah H., Shehata T.M., Morsy M.A. (2019). Nanoemulsion based vehicle for effective ocular delivery of moxifloxacin using experimental design and pharmacokinetic study in rabbits. Pharmaceutics.

[27] Todo H. (2017). Transdermal permeation of drugs in various animal species. Pharmaceutics.

[28] Van Ravenzwaay B., Leibold E. (2004). A comparison between in vitro rat and human and in vivo rat skin absorption studies. Hum. Exp. Toxicol..

[29] Elmataeeshy M.E., Sokar M.S., Bahey-El-Din M., Shaker D.S. (2018). Enhanced transdermal permeability of Terbinafine through novel nanoemulgel formulation; Development, in vitro and in vivo characterization. FJPS.

[30] Lakshmi P., Mounika K., Saroja C. (2014). Transdermal permeation enhancement of lamotrigine using terpenes. J. Pharma Care Health Sys..

[31] Si S., Swain S., Kanungo S., Gupta R. (2006). Preparation and evaluation of gels from gum of Moringa oleifera. Indian J. Pharm. Sci..

[32] Hazrati M., Mehrabani D., Japoni A., Montasery H., Azarpira N., Hamidian-Shirazi A., Tanideh N. (2010). Effect of honey on healing of Pseudomonas aeruginosa infected burn wounds in rat. Appl. Anim. Res..

[33] Ala S., Alvandipour M., Saeedi M., Hamidian M., Shiva A., Rahmani N., Faramarzi F. (2017). Effects of topical atorvastatin (2%) on posthemorrhoidectomy pain and wound healing: A randomized double-blind placebo-controlled clinical trial. World J. Surg..

[34] Shete G., Puri V., Kumar L., Bansal A.K. (2010). Solid state characterization of commercial crystalline and amorphous atorvastatin calcium samples. AAPS Pharm. Sci. Tech..

[35] Palanisamy M., James A., Khanam J. (2016). Atorvastatin–cyclodextrin systems: Physiochemical and biopharmaceutical evaluation. J. Drug Deliv. Sci. Technol..

[36] Sultana S.S., Swapna G., Lakshmi G.S.S., Swathi S., Jyothi G.N., Devi A.S. (2016). Formulation and evaluation of herbal emulgel of Lantana camara leaves extract for wound healing activity in diabetic rats. Indo Am. J. Pharm. Res..

[37] Pandit N.K., Wang D. (1998). Salt effects on the diffusion and release rate of propranolol from poloxamer 407 gels. Int. J. Pharm..

[38] Aly U.F. (2012). Preparation and evaluation of novel topical gel preparations for wound healing in diabetics. Int. J. Pharm. Pharm. Sci..

[39] Majithiya R.J., Ghosh P.K., Umrethia M.L., Murthy R.S. (2006). Thermoreversible-mucoadhesive gel for nasal delivery of sumatriptan. AAPS Pharm. Sci. Tech..

[40] El-Setouhy D.A., Ahmed El-Ashmony S.M. (2010). Ketorolac trometamol topical formulations: Release behaviour, physical characterization, skin permeation, efficacy and gastric safety. J. Pharm. Pharmacol..

[41] Eid A.M., El-Enshasy H.A., Aziz R., Elmarzugi N.A. (2014). Preparation, characterization and anti-inflammatory activity of Swietenia macrophylla nanoemulgel. J. Nanomed. Nanotechnol..

[42] Dhawan B., Aggarwal G., Harikumar S. (2014). Enhanced transdermal permeability of piroxicam through novel nanoemulgel formulation. Int. J. Pharm. Investig..

[43] Rai V.K., Mishra N., Yadav K.S., Yadav N.P. (2018). Nanoemulsion as pharmaceutical carrier for dermal and transdermal drug delivery: Formulation development, stability issues, basic considerations and applications. J. Control. Release.

[44] Syamala U. (2013). Development & optimization of allyl amine antifungal nanoemulgel using 23 factorial design: For the treatment of tinea pedis. Eur. Sci. J..

[45] Ahmad J., Gautam A., Komath S., Bano M., Garg A., Jain K. (2019). Topical nano-emulgel for skin disorders: Formulation approach and characterization. Recent Pat. Antiinfect. Drug Discov..

[46] Lu W.-C., Chiang B.-H., Huang D.-W., Li P.-H. (2014). Skin permeation of D-limonene-based nanoemulsions as a transdermal carrier prepared by ultrasonic emulsification. Ultrason. Sonochem..

[47] Schiefelbein D., Goren I., Fisslthaler B., Schmidt H., Geisslinger G., Pfeilschifter J., Frank S. (2008). Biphasic regulation of HMG-CoA reductase expression and activity during wound healing and its functional role in the control of keratinocyte angiogenic and proliferative responses. J. Biol. Chem..

[48] Pandya V.M., Patel J.K., Patel D.J. (2011). Formulation, optimization and characterization of simvastatin nanosuspension prepared by nanoprecipitation technique. Pharm. Lett..

[49] Hamdan S., Pastar I., Drakulich S., Dikici E., Tomic-Canic M., Deo S., Daunert S. (2017). Nanotechnology-driven therapeutic interventions in wound healing: Potential uses and applications. ACS Cent. Sci..

